# Estimating and Correcting for Off-Target Cellular Contamination in Brain Cell Type Specific RNA-Seq Data

**DOI:** 10.3389/fnmol.2021.637143

**Published:** 2021-03-03

**Authors:** Jordan Sicherman, Dwight F. Newton, Paul Pavlidis, Etienne Sibille, Shreejoy J. Tripathy

**Affiliations:** ^1^Bioinformatics Graduate Program, University of British Columbia, Vancouver, BC, Canada; ^2^Michael Smith Laboratories, University of British Columbia, Vancouver, BC, Canada; ^3^Department of Pharmacology and Toxicology, University of Toronto, Toronto, ON, Canada; ^4^Centre for Addiction and Mental Health, Campbell Family Mental Health Research Institute, Toronto, ON, Canada; ^5^Department of Psychiatry, University of British Columbia, Vancouver, BC, Canada; ^6^Djavad Mowafaghian Centre for Brain Health, University of British Columbia, Vancouver, BC, Canada; ^7^Department of Psychiatry, University of Toronto, Toronto, ON, Canada; ^8^Krembil Centre for Neuroinformatics, Centre for Addiction and Mental Health, Toronto, ON, Canada

**Keywords:** brain, RNA-seq, contamination, single cell, LCM-seq, TRAP-seq

## Abstract

Transcriptionally profiling minor cellular populations remains an ongoing challenge in molecular genomics. Single-cell RNA sequencing has provided valuable insights into a number of hypotheses, but practical and analytical challenges have limited its widespread adoption. A similar approach, which we term single-cell type RNA sequencing (sctRNA-seq), involves the enrichment and sequencing of a pool of cells, yielding cell type-level resolution transcriptomes. While this approach offers benefits in terms of mRNA sampling from targeted cell types, it is potentially affected by off-target contamination from surrounding cell types. Here, we leveraged single-cell sequencing datasets to apply a computational approach for estimating and controlling the amount of off-target cell type contamination in sctRNA-seq datasets. In datasets obtained using a number of technologies for cell purification, we found that most sctRNA-seq datasets tended to show some amount of off-target mRNA contamination from surrounding cells. However, using covariates for cellular contamination in downstream differential expression analyses increased the quality of our models for differential expression analysis in case/control comparisons and typically resulted in the discovery of more differentially expressed genes. In general, our method provides a flexible approach for detecting and controlling off-target cell type contamination in sctRNA-seq datasets.

## Introduction

Traditional RNA-sequencing, which occurs on homogenized bulk tissue samples, has made it possible to have a global view on the entire transcriptome of a great many tissues and species with relative ease and affordability. Although the technology is mature and its analysis is well-documented, it lacks the ability to capture changes in minor cellular populations. For this purpose, newer technologies (single cell RNA-sequencing) are typically used for analysis at the single cell (rather than whole tissue) level (Kim et al., 2015). Although these enable a powerful new view on transcriptomics, they are technically challenging and artifacts such as so-called “dropout” complicate data analysis (Kim et al., 2015). These and high costs remain limiting factors in the widespread adoption of these methods. To balance between the sensitivity of single cell sequencing and the relative affordability of bulk tissue RNA sequencing (RNA-seq), cell type specific transcriptomes are now also being obtained using a variety of technologies and methods (Kim et al., 2015; Yuan et al., 2017). These methods typically involve the enrichment of a specific cell type of interest by morphological or fluorescence-based approaches, followed by the sequencing of a pool of these cells, usually resulting in one pooled cell type group per sample. They are collectively termed single cell type RNA-seq (sctRNA-seq).

There are a number of advantages of sctRNA-seq compared to bulk tissue RNA-seq or even single cell RNA-seq (scRNA-seq). Relative to bulk RNA-seq, sctRNA-seq (and scRNA-seq) can reveal changes in gene expression in minor or rare cell types, or cell types of *a priori* interest, which may obscured by changes in more prevalent cellular populations in traditional expression profiling methods (Hwang et al., 2018). Relative to scRNA-seq, sctRNA-seq affords the choice to sequence mRNA from pools of cells to a greater depth, which could greatly simplify the cost for collection, analysis, and interpretation of these data. Another important difference is that with sctRNA-seq (as compared to scRNA-seq), cell type labels are known *a priori* rather than assigned by cluster associations. For more information on the considerations and differences between these methods, we refer readers to (Hwang et al., 2018; Chen et al., 2019).

Laser capture microdissection coupled with RNA-sequencing (LCM-seq) is one such technology for sctRNA-seq. In LCM-seq, a thin slice of tissue is mounted under a high power microscope with the cells of interest visibly labeled for targeted excision by a laser and collected for sequencing (Bonner et al., 1997; Kummari et al., 2015). There are a number of approaches for labeling cells, including using transgenic animals or through the use of fluorescence *in situ* hybridization (Progatzky et al., 2013). LCM-seq differs from other methods for sctRNA-seq, including Fluorescence Assisted Cell Sorted (FACS) and immunopurification-based methods such as Translating Ribosome Affinity Purification (TRAP-seq), in that it affords the ability to visually target cells *in situ* prior to capture. LCM-seq has been widely adopted in the context of cell type specific profiling, including in motor neurons (Bouçanova et al., 2020; Harjuhaahto et al., 2020; Nizzardo et al., 2020), enterocytes (Moor et al., 2018), cortical (Pereira et al., 2017) and hippocampal neurons (Deng et al., 2019), and cortical interneurons (Shukla et al., 2019; Newton et al., 2020).

**Figure 1:**
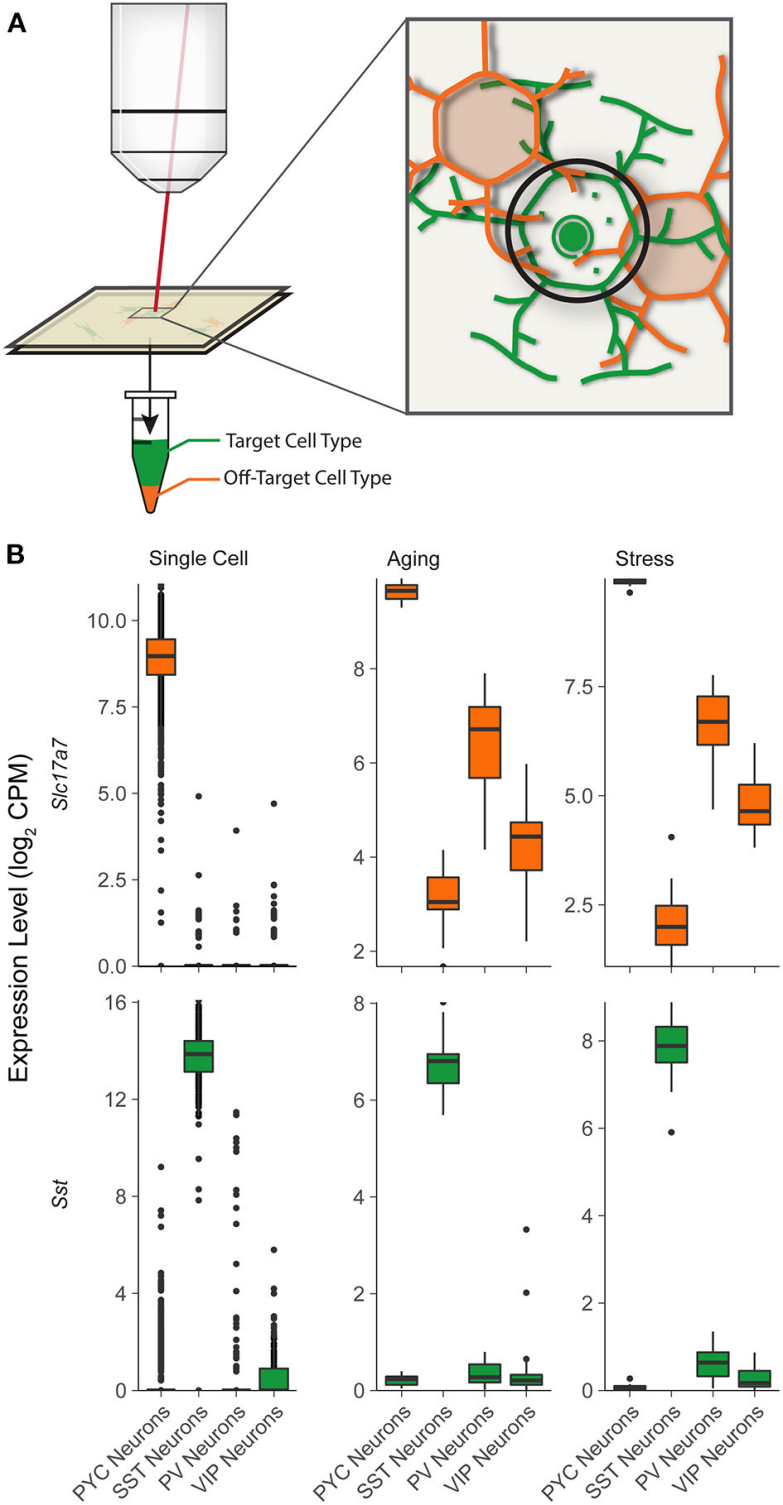
Illustration for potential off-target cellular contamination in single-cell type RNA-seq studies. **(A)** A schematic showing cell targeted mRNA sampling in LCM-seq, illustrating targeted sampling of specific cells (green) along with the potential for undesired sampling from surrounding cells (orange). In this case, the desired SST interneuron (green) will be microdissected by cutting around the black path. In this area, processes of the orange cell (orange) lay above and below the targeted cell (green). **(B)** Expression levels of characteristic cell type specific marker genes for pyramidal cells (*Slc17a7*) and SST interneurons (*Sst*) in “Single Cell” (single cell Allen Brain Institute reference data) and two LCM-Seq experiments: “Aging” (GSE119183) and “Stress” (GSE145521). Note the presence of relatively high levels of *Slc17a7* in samples from SST, PV, and VIP interneurons in the LCM-Seq Aging and Stress datasets, indicating likely off-target contamination.

One potential challenge with LCM-seq is that mRNA from interfering cell bodies and processes may be microdissected along with the cell type of interest (Okaty et al., 2011; Kummari et al., 2015; Rocco et al., 2017). This phenomenon, which we term off-target cell type contamination, may arise in part due to physical overlap or close apposition by surrounding cells or long-range processes (illustrated schematically in Figure 1A). Off-target cellular contamination is especially relevant in brain tissue, in which diverse populations of distinct cell types are densely packed and surrounded by a myriad of other cell types (Kummari et al., 2015; Tremblay et al., 2016). While this potential issue has been noticed previously in the context of microarray data (Okaty et al., 2011) and in other data types, including Patch-seq (Tripathy et al., 2018), to our knowledge there is currently no approach that attempts to systematically identify this issue across sctRNA-seq datasets and assess its potential impact on downstream analyses.

In order to gain a deeper understanding about how off-target cellular contamination might affect data analysis in sctRNA-seq datasets, we applied a computational method to estimate the extent of off-target cellular contamination in sctRNA-seq datasets. Our approach relies upon the availability of high-quality scRNA-seq data as a reference for comparisons. The output of the analysis is a set of coefficients that estimate, on a per-sample basis, the degree of off-target cellular contamination per cell type in the surrounding tissue. We present two primary use-cases for these cellular contamination estimates in the context of brain sctRNA-seq analysis. The first is in simply assessing the quality and purity of collected sctRNA-seq derived transcriptomes. The second is in using these coefficients in the context of condition-specific differential expression analyses to help correct for the confounds of sample-to-sample and across-condition variances in sample quality.

## Materials and Methods

### Access and Pre-processing of Single Cell Type RNA-seq Data

Raw reads for each LCM-seq library were downloaded from GEO (GSE119183 and GSE145521) and aligned using STAR (v2.7.5) (https://github.com/alexdobin/STAR) to the mouse genome build mm10_ensembl98. The following options were used: —*outSAMtype BAM SortedByCoordinate—quantMode GeneCounts—outReadsUnmapped Fastx—limitBAMsortRAM 10000000000—outFilterMultimapNmax 1*. PCR duplicates were removed by running STAR on the original output with the options: —*bamRemoveDuplicatesType UniqueIdentical—runMode inputAlignmentsFromBAM*. Only reads that aligned uniquely were retained. After mapping, quantification was performed by aligning to exons and introns separately. Other sctRNA-seq data were downloaded as filtered count matrices from Gemma (Lim et al., 2020).

### Access of Single Cell Reference Dataset

As a reference, we used single cell transcriptomic profiles from the Allen Institute for Brain Sciences from multiple neocortical regions and the hippocampus from mouse brain samples. To our knowledge, this is the largest and highest quality publicly available data of this type. Single cell transcriptomes were downloaded from the Brain Map Portal (https://portal.brain-map.org/atlases-and-data/rnaseq) for mouse whole cortex and hippocampus SMART-seq (2019) as cell metadata and gene expression matrices for introns and exons separately.

### Comparison of Single Cell Type RNA-seq Data to Single Cell Reference Data

Here, we give a general overview of the analytical workflow before going into further detail below.

1) We generate a list of marker genes for each cell type using the single cell reference data.
2) We then summarize the single cell reference data to the level of cell types by taking the average gene-wise expression level of all cells within each type.
3) We evaluate the similarity of sctRNA-seq data to the summarized scRNA-seq data. Similarity is scored as the Pearson correlation of the expression levels of the marker genes in the test sctRNA-seq data to the summarized scRNA-seq expression profiles generated in 2). This key step generalizes and builds on a method described by Tripathy et al. (2018) in which a scalar contamination index is generated using similar assumptions.
4) To contextualize these scores against the expected biological similarities, we scale “contamination coefficients” based on the average correlation of a subsample of reference single cells to the collapsed reference data generated in 2).

#### Determination of Cell Type Specific Marker Genes

Seurat's (v3.1.5) *FindAllMarkers* function was used with MAST (v1.12.0) differential expression testing to generate a robust set of cell type specific marker genes on the single cell Allen Brain data (Stuart et al., 2019). MAST is well-suited for this task as it utilizes a hurdle model tailored for single cell RNA-seq (scRNA-seq) data (Finak et al., 2015). To ensure our cell type marker genes exhibit a relatively binary (on/off) expression pattern between the cell type of interest and all other cell types, we applied stringent settings such that there must be at least a 40% difference in the fraction of detection between the two populations. Only genes that exhibited at least a two-fold difference in expression between populations (at an FDR of 0.01) were retained for downstream analysis (Supplementary Table 1).

#### Calculation of Cluster Centroids

To facilitate pairwise gene correlations, we collapsed the cell-level gene expression matrices into per-cluster truncated interquartile means of the log_2_ counts-per-million (i.e., after excluding the lowest and highest 25% of normalized gene counts) of each gene.

#### Per-sample Correlation and Normalization Scaling

The Pearson correlation of the log_2_ normalized marker gene expression levels of each sample in the test data (enriched for a single cell type) to the expression centroids of the reference cell types (defined above) is used as a proxy for sample purity. This can be expressed mathematically as [cor(log_2_(1 + CPM)_markers, test_, log_2_(mean(1 + CPM))_markers, reference_]. It is calculated on the expression of marker genes only (as opposed to the whole gene-set) because most of the variance in their expression is expected to be due to presence of off-target cell types rather than true biological differences. To reduce the impact of outlying marker genes and to estimate the confidence interval of these correlations, we randomly subsample a smaller fraction (60%) of the total marker genes (with an equal number of marker genes per cell type), calculate the correlation of each sample to every reference cell type, and repeat for 10,000 iterations. We then use the average correlation across all iterations as the consensus correlation coefficient and retain the entire set for downstream statistical analysis.

These coefficients are more readily interpretable after they are scaled by the expected biological correlation. For instance, a sample enriched for cell type *A* whose marker gene expression profile correlates with the reference cell type *B* at a Pearson's r of 0.6 may naively seem to be unexpectedly highly correlated, but if the correlation of a *true* cell of type *A* to the same reference is also 0.6, the sample is actually behaving exactly as expected.

Using this approach, to obtain estimates on this expected level of biological similarity between cell types, we selected a random subsample of single cells in the reference data and correlated their expression profiles against the centroids of the same reference data (as described above). Since each cell in the reference data is annotated with its “true” cell type, this yielded a set of expected correlation coefficients for each cell type to every cell type. Scaling the correlation coefficients obtained on the test data to the respective set of expected coefficients thus results in a fractional score where the test data correlates at *x* times the expected value, producing easily interpretable purity scores.

### Differential Expression Analysis

DESeq2 (v1.26.0) was used for differential expression analysis, pre-filtering genes that had zero counts in more than half of the samples per condition to ensure analysis was restricted to those which we had sufficient power to call differential expression (Love et al., 2014). An FDR threshold of 0.1 was used to select differentially expressed genes (DEGs). We performed this analysis both with and without correlation coefficients (for the top four off-target cell types) reflecting off-target contamination scores. To assess the amount of overlap between these two analyses, we generated hypergeometric *p*-values using the RRHO (v1.26.0) package in R (Rosenblatt and Stein, 2020). To more objectively evaluate which model (whether including contamination coefficients or not) better fits these data, we calculated the Akaike information criterion (AIC) for each gene's model fit. Under this estimator, lower scores correspond to less information loss, and thus, the model with lowest gene-wise AIC value fits the data the best.

### Synthetic Data

We generated synthetic cell type specific gene expression data according to methods outlined in Soneson and Delorenzi (2013) for 20,000 synthetic genes with 3,000 genes differentially expressed. Cellular identities were added to these “pure” synthetic clusters by appending the marker gene expression profiles corresponding to the reference data centroids outlined above (such that each pure cluster would correlate with its reference cell types with *r*^2^ = 1). Contamination by other synthetic clusters was then simulated by biasing the expression profiles toward the expression level of other pure clusters by a predefined fraction with a set amount of inter- and intra-cluster noise. We also modeled a confounding effect of contamination by biasing high contamination toward one condition (and low contamination in the other) for each cluster.

## Results

### Illustration of Off-Target Cellular Contamination

As an initial examination into potential off-target contamination in sctRNA-seq datasets (shown schematically in Figure 1A), we reanalyzed two recent LCM-seq datasets collected from the mouse brain, where fluorescence *in situ* hybridization (FISH) was used to label specific neuronal cell types prior to LCM-based cellular isolation (Shukla et al., 2019; Newton et al., 2020). We contrasted these datasets with an additional dataset made available by the Allen Institute for Brain Sciences, where cells were first dissociated and sorted prior to scRNA-seq, presumed to likely be free from off-target cellular contamination (Tasic et al., 2018).

We first examined expression of gene expression markers for various cell types in the reference scRNA-seq dataset. We found virtually no detected expression of gene expression markers for off-target cell types in the reference mouse scRNA-seq dataset. For example, pyramidal cells showed strong expression of markers known to be expressed in pyramidal cells, such as *Slc17a7* or *VGLUT1* (Figure 1B, top row). Pyramidal cells sampled by scRNA-seq also showed little to no expression of markers of various subtypes of interneurons, *Pvalb, Sst, or Vip* (markers of PV, SST, and VIP GABAergic interneurons, respectively).

In contrast, in the LCM-seq based sctRNA-seq datasets, we observed considerable expression of the pyramidal cell marker, *Slc17a7* in each of the PV, SST, and VIP interneuronal types, suggesting off-target contamination by pyramidal cells. Contrastingly, we did not observe much or any expression of *Sst* mRNA in pyramidal cells, PV, or VIP interneurons, indicating little off-target contamination contributed by SST interneurons in other cell types. One explanation for these observations is that since pyramidal cells are physically larger, more numerous, and have a greater extent of projections throughout the entire tissue than interneurons (Erö et al., 2018), they are more likely to contribute off-target contamination than interneuron cell types.

### Brain LCM-Seq Data Is Subject to Measurable Levels of Contamination

We used this analytical approach to quantify the levels of off-target cellular contamination in publicly available LCM-seq datasets. In brief, this approach seeks to quantify the extent of off-target cell type contamination by scoring each sample's transcriptional similarity against each of the cell types available in the single cell reference data. Specifically, for each sctRNA-seq sample, the method yields off-target contamination coefficients per tested cell type in the scRNA-seq reference data. These coefficients are scaled such that values at or near 1.0 indicate no detectable off-target contamination and values >1.0 indicates off-target cellular contamination (see section Materials and Methods for further details).

**Figure 2:**
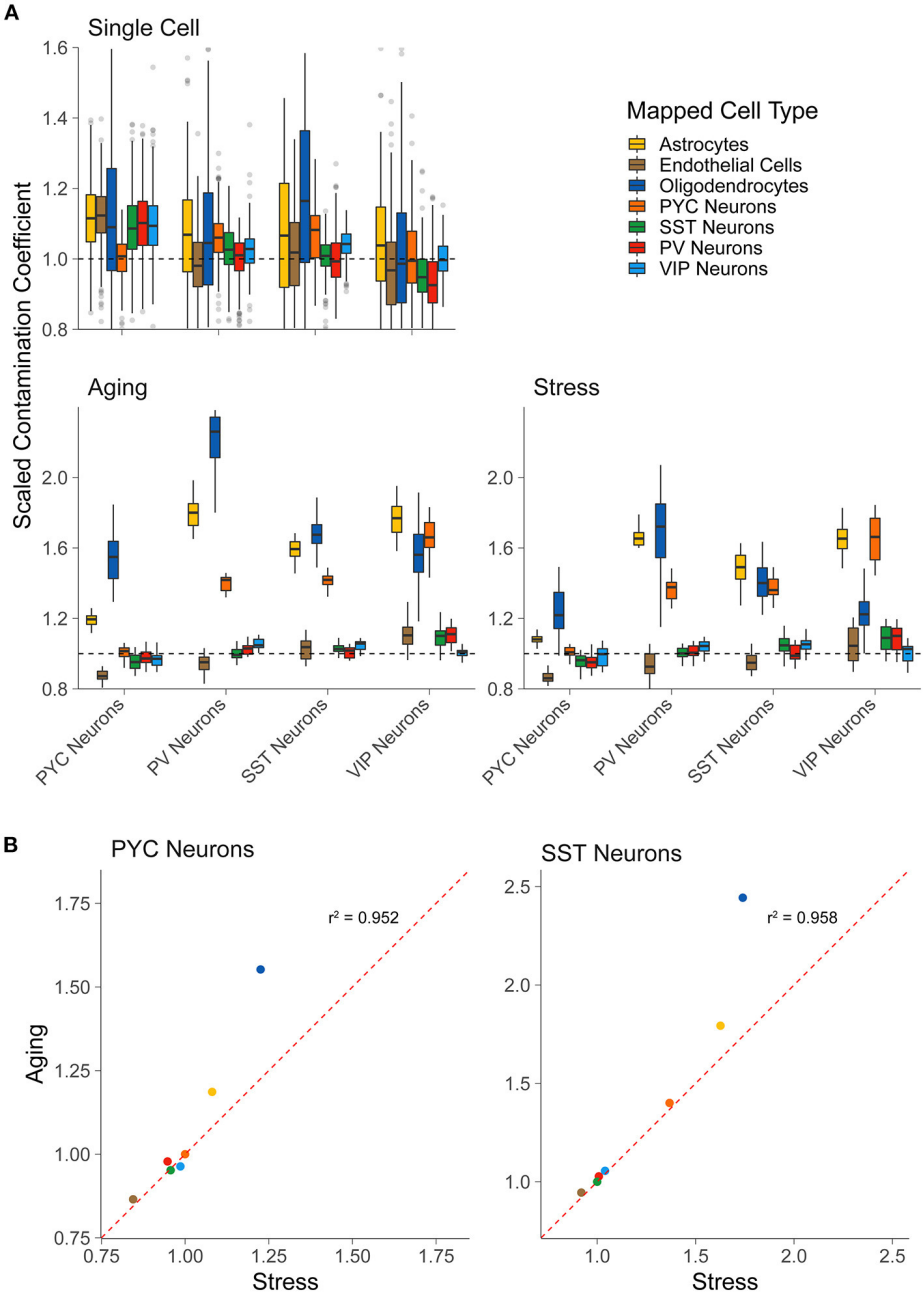
LCM-seq but not scRNA-seq shows off-target cell-type marker expression. **(A)** Scaled contamination coefficients for an alternative scRNA-seq dataset (Tasic et al., 2016) that was not used previously as reference data (top row), the LCM-seq Aging dataset, and the LCM-seq Stress dataset (bottom row, left to right). Y-axis values are scaled to 1.0, which indicates the expected amount of off-target cellular marker expression if the sample was behaving exactly as expected for its “actual” cell type [based on the scRNA-seq reference data from Tasic et al. (2018)]. The “actual” cell type (for which the sample was enriched) is on the x-axis and the groups represent the reference cell type from single cell reference data. **(B)** Scatterplots for the scaled contamination coefficients in the Stress and Aging LCM-seq datasets for samples enriched for pyramidal cells (PYC Neurons) and SST interneurons (SST Neurons). Each point reflects the off-target cell type group averages shown in **(A)**.

As a control, we first applied our approach to calculate contamination coefficients in an separate scRNA-seq dataset (Tasic et al., 2016), collected using similar methodologies as our reference scRNA-seq dataset (Tasic et al., 2018). We found that each of the derived contamination coefficients in this separate scRNA-seq dataset were centered at or near 1.0, indicating no or little contamination. However, we noticed in some cases, such as astrocyte contamination coefficients observed in pyramidal cells, that the off-target contamination coefficients modestly diverged from the expected value of 1.0 (Figure 2A top row). Such minor deviations could be in part due to true variability (in data quality, biological noise, etc.) between the two scRNA-seq datasets, but may also demonstrate some uncertainty in the coefficients.

Applying our approach to the two LCM-seq based sctRNA-seq datasets, “Stress” and “Aging,” we found that each sctRNA-seq sample demonstrated some cellular contamination due to several off-target cell types (Figure 2A, bottom row). For example, we found that each of the interneuron subtypes in the LCM-seq datasets displayed a degree of contamination from pyramidal cells, supporting the qualitative analysis of *Slc17a7* gene expression shown in Figure 1B. We also observed off-target contamination by oligodendrocytes and astrocytes in each profiled cell type, including pyramidal cells and SST, PV, and VIP interneurons. Encouragingly, off-target contamination due to interneuronal cell types observed in pyramidal cell samples (or in other interneuronal types) appears low, again supporting the qualitative observations shown in Figure 1B. Finally, we observed that pyramidal cell sctRNA-seq samples showed less off-target contamination than each of the profiled interneuronal cell types, supporting the notion that these larger neurons are more easily separated from contaminating cell types than the smaller interneurons.

As a final point, we noted that the degree of off-target cellular contamination is similar between cell types sampled from the two LCM-seq datasets in our analysis (Figure 2B). For example, SST interneuron samples tended to show the most contamination due to astrocytes, pyramidal cells, and oligodendrocytes, regardless of the dataset of origin. This observation, albeit anecdotal, is interesting as it might represent shared but replicable sampling limitations using the LCM-seq methodology.

### Contamination Coefficients Improves Differential Expression Analysis in Synthetic Data

We next assessed the extent to which off target-cellular contamination might affect the results of downstream analyses, such as differential expression analyses in the context of a condition, such as aging or stress (Shukla et al., 2019; Newton et al., 2020). To first understand how various factors might impact differential expression analyses in sctRNA-seq, we used a modeling approach where we simulated multiple synthetic sctRNA-seq datasets with varying degrees of off-target cellular contamination and magnitude of differential expression signal. We further modeled the effect of contamination that is confounded with the condition or contrast of interest, specifically asking how this affects the accuracy or validity of downstream differential expressed genes (DEGs). We evaluated the efficacy of our contamination coefficients in improving differential expression analysis by including them as covariates in the differential expression linear model and calculating the difference in area under the resulting receiver operator characteristic curve (AUROC), as well as the difference in false positive and negative calls.

Our simulations revealed that, in general, as the amount of off-target contamination and degree of confoundedness of contamination with the condition of interest increases, including the coefficients improves differential expression analysis. We observed this effect both by an increase in AUROC and in a reduction in false positive calls without a corresponding increase in false negative calls. This is true regardless of the mean effect size (Supplementary Figure 1, top row). In addition, in cases of no off-target contamination, incorporating contamination coefficients had a negligible effect on the analysis (Table 1). Together these analyses on synthetic datasets suggest that these off-target contamination coefficients will improve differential expression analyses in real sctRNA-seq datasets.

**Table 1:** Summary of simulation experiments.

**Contamination**	**Confound**	**FPF**		**FNF**		**AUROC**	
		**Uncorrected**	**Corrected**	**Uncorrected**	**Corrected**	**Uncorrected**	**Corrected**
None	None	12%	2%	10%	3%	0.982	0.981
Low	Low	12%	3%	8%	3%	0.863	0.899
High	Low	29%	3%	15%	3%	0.799	0.902
High	High	79%	5%	33%	14%	0.618	0.677

*Summary of synthetic data simulations varying contamination (either low: 5%, high: 20%, or none: 0% by the contaminating group) and confoundedness (either low: 20%, high: 100%, or none: 0%). False positive/false negative fractions (FPF/FNF) are given as the proportion of calls at FDR < 0.1. Note that lower false positive and negative rates are better*.

#### Accounting for Cellular Contamination Improves Detection of Differentially Expressed Genes in Real-World sctRNA-seq Datasets

We next used our derived contamination coefficients in the context of differential expression analyses in real-world LCM-seq datasets. We performed differential expression analysis on the “Aging” and “Stress” LCM-seq datasets to assess genes whose expression levels are changing in the context of aging and stress.

We first evaluated the efficacy of incorporating contamination coefficients using a statistical information criterion approach. Specifically, since differential expression analysis involves fitting linear models to the gene expression data, we assessed whether including contamination coefficients as a covariate in the model improves the fit of these models on each gene individually (see section Materials and Methods). Given a pair of possible models, the preferred one is the one that yields the smaller AIC value. We found that models with contamination coefficients were estimated to minimize the information loss (represented by smaller AIC values on a per-gene basis) 64 and 67% of the time in the Aging and Stress datasets, respectively, compared to the minimal models. This is significantly better than 50% which would be selected by random chance (i.e., if the model pairs fit the data approximately equally well). These indicate that including the contamination coefficients likely leads to a better model most of the time.

**Figure 3:**
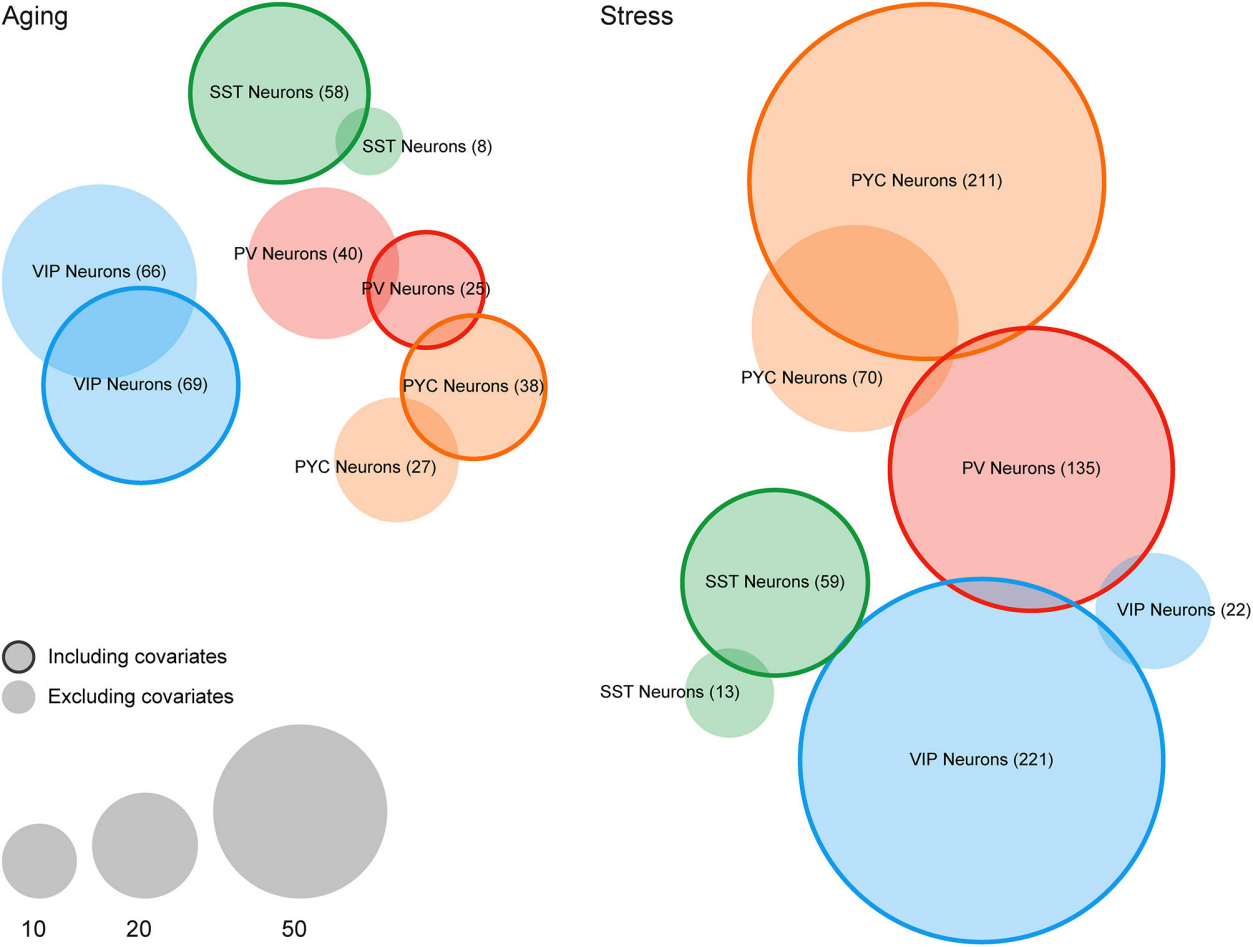
Differential expression analysis of case/condition LCM-seq datasets with and without including covariates for off-target cellular contamination. Venn diagrams indicating the number of differentially expressed genes (FDR < 0.1) in the Aging and Stress datasets. Non-outlined circles indicate counts of differentially expressed genes without including cellular contamination covariates and outlined circles indicate counts following inclusion of covariates. Empty sets (i.e., PV Neurons in the Stress dataset) are not shown.

In terms of differential expression events at a particular statistical threshold, we found that many more genes were called at FDR < 0.1 after including covariates for cellular contamination in our linear models (see section Materials and Methods). For example, for SST interneurons, we detected eight genes differentially expressed in the Aging dataset without inclusion of contamination coefficients but detected 58 genes after the inclusion of covariates (Figure 3). In the Stress dataset, we noticed a similar, but more profound trend, with 0 and 22 genes detected as differentially expressed among PV and VIP interneurons (respectively) without covariates, but 135 and 221 genes after inclusion of covariates. In only one instance, PV Neurons in the Aging dataset, were fewer genes called as differentially expressed after including contamination coefficients. A complete list of differentially expressed genes derived from both LCM-seq datasets is provided Supplementary Table 2.

Of note, the global rankings of gene differential expression by *p*-value remain quite similar before and after cellular contamination covariate inclusion (minimum hypergeometric *p*-value < 0.001 for all cell types). This suggests that while inclusion of these covariates may result in somewhat more DEGs passing a statistical threshold, their effect might nevertheless be moderate. In general, we predict that the effect of including contamination coefficients will depend on a number of factors, including the strength of true expression differences relative to the sample-to-sample variance caused by off-target contamination.

### Off-Target Cellular Contamination Is Prevalent Across Multiple Methodologies of Cell Purification for sctRNA-seq

As a final analysis, we were interested in evaluating how prevalent off-target cellular contamination might be in datasets sampled using alternative strategies for sctRNA-seq. Focusing on pyramidal cell sctRNA-seq datasets sampled using a number of methodologies (see Table 2), we re-analyzed two TRAP-seq datasets [GSE141337 (Cocaine 1) and GSE141464 (Cocaine 2)], which includes samples of S100a10- and Glt25d2-labeled pyramidal cells from the mouse cerebral cortex from which mRNAs from translating ribosomes were immunopurified and sequenced. We also re-analyzed Hipposeq, which includes samples from pyramidal cells specific to hippocampal areas CA1, CA2, and CA3 (Cembrowski et al., 2016). Samples from this data were generated by dissociating and manually sorting fluorescently-labeled cells, which is generally thought to generate relatively pure cell type pools as compared to other sctRNA-seq methodologies (Hempel et al., 2007).

**Table 2:** Datasets analyzed in this work.

**GSE**	**DOI**	**Type**	**Description**
GSE119183	10.1016/j.biopsych.2018.09.019	LCM-seq	Aging
GSE145521	10.1101/2020.08.18.249995	LCM-seq	Stress
GSE141337	*Unpublished*	TRAP-seq	Cocaine 1
GSE141464	*Unpublished*	TRAP-seq	Cocaine 2
GSE115746	10.1038/s41586-018-0654-5	scRNA-seq	Reference data
GSE71585	10.1038/nn.4216	scRNA-seq	Single cell data

*A summary of datasets used in this paper. In the manuscript, they are referred to by the name given in the description column*.

**Figure 4:**
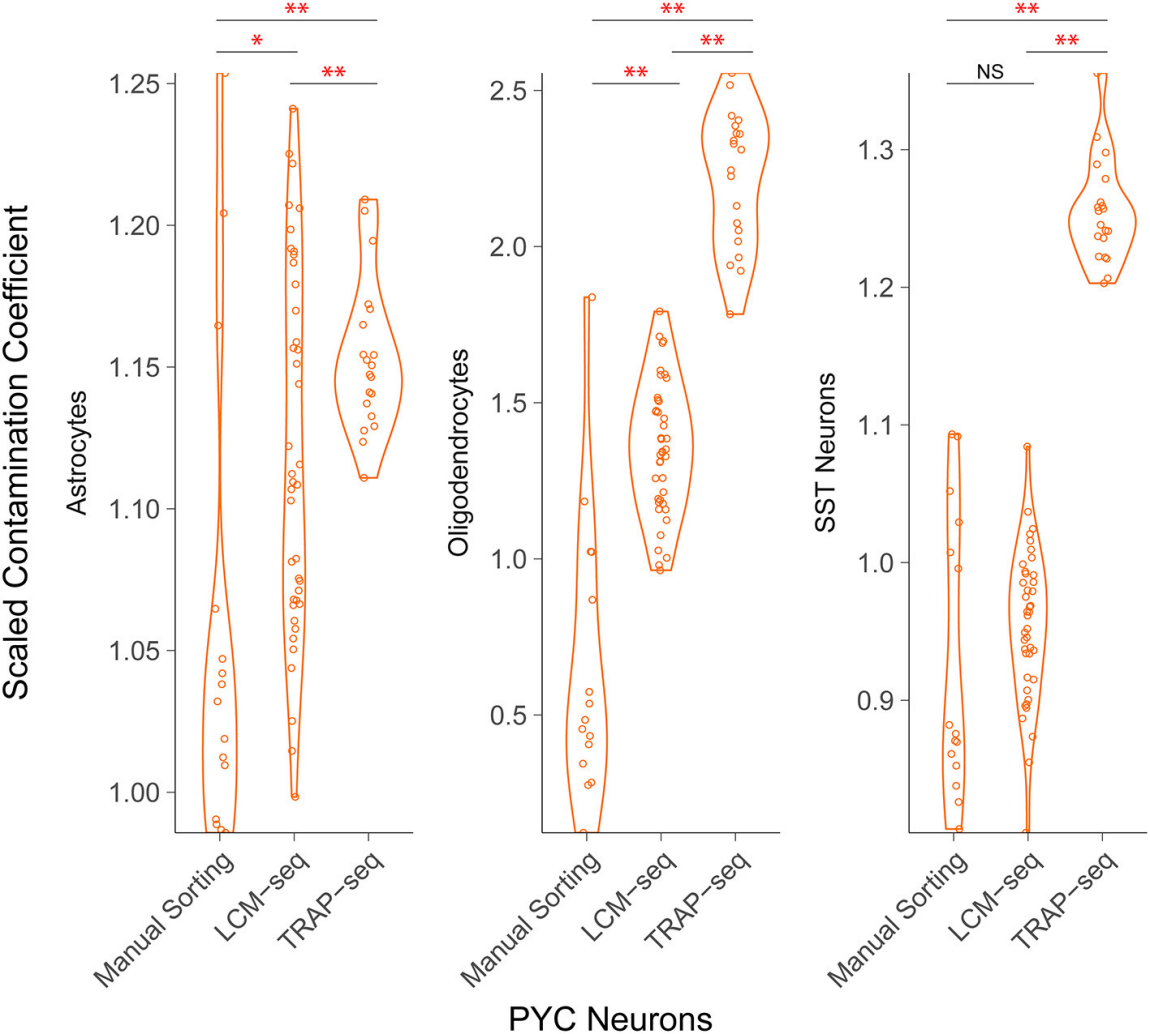
Comparison of off-target contamination across cell purification methodologies. Estimated relative amount of off-target cellular contamination among different methodologies. Each point represents a single sample (a cell pool). Both LCM-seq datasets (Aging and Stress) were grouped, as well as both TRAP-seq datasets (Cocaine 1 and 2). The difference in mean contamination coefficients is statistically significant for most comparisons *p < 0.05, **p < 0.01.

We found that among the sctRNA-seq methodologies, fluorescence-based manual sorting exhibited the least off-target contamination followed by LCM-seq and then TRAP-seq (Figure 4). For example, we found that among pyramidal cells sampled across each of these technologies, we found off-target contamination from oligodendrocytes yielded mean scaled contamination coefficients of 0.65, 1.35, and 2.21 in manually sorted cells, LCM-seq, and TRAP-seq, respectively. These differences were significant in all pairwise comparisons (*p* < 10^−9^ and 10^−16^ for manual sorting vs. LCM-seq and LCM vs. TRAP-seq, respectively). Across technologies, we found that non-neuronal cell types (as opposed to interneurons) contributed the greatest amount of off-target contamination.

## Discussion

In this work, we applied an approach for characterizing off-target cellular contamination in RNA-seq-based datasets of pooled cell types (sctRNA-seq datasets). By comparing these sctRNA-seq samples to analogous high-purity scRNA-seq datasets, we were able to derive a series of contamination coefficients that yield an estimated amount of contamination for each off-target cell type. We note that using markers derived from single cell RNA-seq data and applying them to RNA-seq datasets to derive cell type-specific insights is commonly done in the context of cellular deconvolution applications for bulk and spatial transcriptomics datasets (Wang et al., 2019; Elosua et al., 2021), including methods developed by our group previously (Mancarci et al., 2017; Toker et al., 2018). Here, in the context of sctRNA-seq datasets, such coefficients help contextualize the cellular sources and relative amounts of off-target cellular contamination. They can be used to help correct for sample-to-sample differences in purity in downstream analyses. Further, by applying our approach to multiple sctRNA-seq datasets collected using different methodologies for cell type-specific enrichment (LCM, TRAP, and manual purification), we were able to qualitatively compare how these different methodologies compare in terms of sample purity. Though our applications here were focused on sctRNA-seq datasets collected from the mouse neocortex and hippocampus, this method can in principle be applied to any species or tissue.

We found that pyramidal cells and non-neuronal cells, namely oligodendrocytes and astrocytes, were among the greatest contributors to off-target contamination in each dataset and cell purification methodology we re-analyzed. Across methodologies, we observed that manual sorting appears to show the least apparent off-target contamination, followed by LCM-seq and then by TRAP-seq, consistent with previous reports based on microarray datasets (Okaty et al., 2011). We reason that to a large extent, this cellular contamination is likely to reflect underlying biology. For example, apparent pyramidal cell contamination in interneuron-specific samples, as we observed here for LCM-seq and we and others observed previously for Patch-seq (Tripathy et al., 2018; Gouwens et al., 2020; Scala et al., 2020), is likely due to pyramidal cells' large size and extensive projections throughout the neocortex. As such, pyramidal cells are more likely to have their mRNA captured inadvertently when sampling non-pyramidal cell types such as interneurons. Interestingly, we found anecdotal evidence that sources of off-target contamination tended to be similar in different datasets sampled using the same methodology despite being collected by different experimenters such as the LCM-seq mouse Aging (Shukla et al., 2019) and Stress (Newton et al., 2020) datasets reported here. Together, these findings indicate that off-target contamination is widely present, but of overall modest size (relative to expectations based on single cell reference data), and likely an unavoidable consequence of various methodologies for sctRNA-seq.

Despite the modest effect sizes of contamination associated with single cell type captures, we show analytical improvement when correcting for off-target contamination in the context of group-level modeling analyses, such as case/control differential expression analyses. We note that this is just one possible use of such covariates: they may also be used in other ways, including to remove samples with outlying levels of contamination, as covariates in analyses such as limma (Ritchie et al., 2015) or ComBat (Johnson et al., 2007) to normalize gene-level read counts for contamination, etc. Applying our approach initially within the context of a simulation-based framework, we found that incorporating contamination coefficients was effective in improving true calls of differentially expressed genes without a corresponding increase in false calls. This approach was also effective when contamination was confounded with or biased toward a particular group (e.g., greater contamination in the “disease” group). Applying our approach to real-world LCM-seq datasets, we found a greater number of differentially expressed genes per cell type. Importantly, given that the models that included our coefficients provided a statistically improved fit to these data relative to models without them, we further posit that correcting for off-target cellular contamination is likely to improve the quality of differentially expressed gene calls (rather than simply increasing the number thereof). However, we note that without experimental validation, we do not know which genes are truly differentially expressed between condition and control, making it difficult to rigorously evaluate the accuracy and validity of this approach.

Based on our simulation and real-data analyses, we expect that including covariates for sample-specific off-target contamination will tend to improve such group-level analyses. Nonetheless, correcting for off-target contamination when it is mild and consistent across experimental conditions is of minimal benefit. As we have described, the physical source of off-target contamination is necessarily due to the presence of cellular soma and processes surrounding the cell of interest (see Figure 1). Indeed, many psychiatric and neurological disorders are characterized by altered neuronal and astroglial morphology, which pose potential confounds to the interpretation of LCM and TRAP-seq data. Examples include reduced soma size of hippocampal neurons in major depressive disorder (MDD) (Stockmeier et al., 2004), reduced pyramidal cell dendritic branching in natural aging (Luebke et al., 2015) and MDD (Qiao et al., 2016), reduced astrocyte size and density in MDD, bipolar disorder, and schizophrenia (Cotter et al., 2002), and regionally-varying atrophy and hypertrophy of astrocytes in Alzheimer's disease (Verkhratsky et al., 2019). The morphological changes outlined above represent sources of contamination that are confounded with disease and thus may introduce false differentially expressed genes or mask the true differential expression of others. These kinds of conditions are thus likely to benefit the most from the approach we outline.

The major assumption underlying our approach is that high-purity scRNA-seq datasets can provide a useful proxy for sctRNA-seq samples. This assumption, while reasonable, has a number of limitations. First and foremost, this approach requires the availability of scRNA-seq reference datasets, such as those we used here from the mouse neocortex (Tasic et al., 2018). Second, there may be genuine differences in sctRNA-seq expression profiles relative to scRNA-seq that are not the result of contamination; for example, such differences might arise due to technical factors such as differences in library preparation and collection protocol [which may also include transcriptional differences related to cellular dissociation-based cell stress (Adam et al., 2017)]. Similarly, true biological differences can result in large changes in cell-state, including condition- or disorder-specific transcriptional regulation (Lee and Young, 2013), or cell differentiation. As our approach relies on the quality of cell type-specific marker genes: unsuitable markers (e.g., those that are markers of cell state but not cell type) or those with sufficiently non-binary expression patterns may render the derived contamination coefficients unusable. A related limitation is that as the markers derived here are markers based on scRNA-seq samples from dissociated cell bodies, they may not be entirely reflective mRNA in more distal cellular processes (Glock et al., 2017). Each of these caveats are likely to affect each dataset to varying degrees and we advise that users carefully consider these assumptions prior to the use of these methods.

Despite the fact that some amount of contamination seems to be inevitable by current methodologies, cell type specific RNA sequencing offers several advantages compared to traditional bulk or single cell RNA sequencing. Our approach for estimating sample-to-sample differences in purity provides a simple yet flexible approach to account and control for their effect in downstream analyses, allowing for more meaningful interpretation of sctRNA-seq datasets.

## Data Availability Statement

The original contributions presented in the study are publicly available. The code needed to perform the analyses can be found as an R script online at: https://github.com/jsicherman/ sct-contamination, built on R version 3.6.0 (Planting of a Tree).

## Author Contributions

ST and ES designed the study. ST, PP, and ES supervised the study. ST, PP, ES, and DN provided input on data analysis. JS performed data analysis. JS and ST wrote the manuscript with input from all authors. All authors contributed to the article and approved the submitted version.

## Conflict of Interest

The authors declare that the research was conducted in the absence of any commercial or financial relationships that could be construed as a potential conflict of interest.

## References

[B1] Adam M. Potter A. S. Potter S. S. (2017). Psychrophilic proteases dramatically reduce single-cell RNA-seq artifacts: a molecular atlas of kidney development. Development 144, 3625–3632. 10.1242/dev.151142 28851704 PMC5665481

[B2] Bonner R. F. Emmert-Buck M. Cole K. Pohida T. Chuaqui R. Goldstein S.. (1997). Laser capture microdissection: molecular analysis of tissue. Science 278, 1481–1483. 10.1126/science.278.5342.1481 9411767

[B3] Bouçanova F. Pollmeier G. Sandor K. Urbina C. M. Nijssen J. Médard J.-J.. (2020). Disrupted function of lactate transporter MCT1, but not MCT4, in Schwann cells affects the maintenance of motor end-plate innervation. Glia 69, 124–136. 10.1002/glia.23889 32686211

[B4] Cembrowski M. S. Wang L. Sugino K. Shields B. C. Spruston N. (2016). Hipposeq: a comprehensive RNA-seq database of gene expression in hippocampal principal neurons. eLife 5:e14997. 10.7554/eLife.14997 27113915 PMC4846374

[B5] Chen G. Ning B. Shi T. (2019). Single-cell RNA-seq technologies and related computational data analysis. Front. Genet. 10:317. 10.3389/fgene.2019.00317 31024627 PMC6460256

[B6] Cotter D. Mackay D. Chana G. Beasley C. Landau S. Everall I. P. (2002). Reduced neuronal size and glial cell density in area 9 of the dorsolateral prefrontal cortex in subjects with major depressive disorder. Cereb Cortex 12, 386–394. 10.1093/cercor/12.4.386 11884354

[B7] Deng W. Xing C. David R. Mastroeni D. Ning M. Lo E. H.. (2019). AmpliSeq transcriptome of laser captured neurons from Alzheimer brain: comparison of single cell versus neuron pools. Aging Dis. 10:1146. 10.14336/AD.2019.0225 31788328 PMC6844587

[B8] Elosua M. Nieto P. Mereu E. Gut I. Heyn H. (2021). SPOTlight: seeded NMF regression to deconvolute spatial transcriptomics spots with single-cell transcriptomes. Nucleic Acids Res. 10.1093/nar/gkab043 33544846 PMC8136778

[B9] Erö C. Gewaltig M.-O. Keller D. Markram H. (2018). A cell atlas for the mouse brain. Front. Neuroinform. 12:84. 10.3389/fninf.2018.00084 30546301 PMC6280067

[B10] Finak G. McDavid A. Yajima M. Deng J. Gersuk V. Shalek A. K.. (2015). MAST: a flexible statistical framework for assessing transcriptional changes and characterizing heterogeneity in single-cell RNA sequencing data. Genome Biol. 16:278. 10.1186/s13059-015-0844-5 26653891 PMC4676162

[B11] Glock C. Heumüller M. Schuman E. M. (2017). mRNA transport and local translation in neurons. Curr. Opin. Neurobiol. 45, 169–177. 10.1016/j.conb.2017.05.005 28633045

[B12] Gouwens N. W. Sorensen S. A. Baftizadeh F. Budzillo A. Lee B. R. Jarsky T.. (2020). Integrated Morphoelectric and Transcriptomic Classification of Cortical GABAergic Cells. Cell. 183, 845–847. 10.1016/j.cell.2020.09.057 33186530 PMC7781065

[B13] Harjuhaahto S. Rasila T. S. Molchanova S. M. Woldegebriel R. Kvist J. Konovalova S.. (2020). ALS and Parkinson's disease genes CHCHD10 and CHCHD2 modify synaptic transcriptomes in human iPSC-derived motor neurons. Neurobiol. Dis. 141:104940. 10.1016/j.nbd.2020.104940 32437855

[B14] Hempel C. M. Sugino K. Nelson S. B. (2007). A manual method for the purification of fluorescently labeled neurons from the mammalian brain. Nat. Protoc. 2, 2924–2929. 10.1038/nprot.2007.416 18007629

[B15] Hwang B. Lee J. H. Bang D. (2018). Single-cell RNA sequencing technologies and bioinformatics pipelines. Exp. Mol. Med. 50, 1–14. 10.1038/s12276-018-0071-8 30089861 PMC6082860

[B16] Johnson R. Onwuegbuzie A. Turner L. (2007). Toward a definition of mixed methods research. J. Mix. Methods Res. 1, 112–133. 10.1177/1558689806298224

[B17] Kim T. Lim C.-S. Kaang B.-K. (2015). Cell type-specific gene expression profiling in brain tissue: comparison between TRAP, LCM, and RNA-seq. BMB Rep. 48, 388–394. 10.5483/BMBRep.2015.48.7.218 25603796 PMC4577288

[B18] Kummari E. Guo-Ross S. X. Eells J. B. (2015). Laser capture microdissection–a demonstration of the isolation of individual dopamine neurons and the entire ventral tegmental area. J. Vis. Exp. 96. 10.3791/52336 25742438 PMC4354571

[B19] Lee T. I. Young R. A. (2013). Transcriptional regulation and its misregulation in disease. Cell 152, 1237–1251. 10.1016/j.cell.2013.02.014 23498934 PMC3640494

[B20] Lim N. Tesar S. Belmadani M. Poirier-Morency G. Mancarci B. O. Sicherman J.. (2020). Curation of over 10,000 transcriptomic studies to enable data reuse. bioRxiv 2020.07.13.201442. 10.1101/2020.07.13.201442 PMC7904053 33599246

[B21] Love M. I. Huber W. Anders S. (2014). Moderated estimation of fold change and dispersion for RNA-seq data with DESeq2. Genome Biol. 15:550. 10.1186/s13059-014-0550-8 25516281 PMC4302049

[B22] Luebke J. I. Medalla M. Amatrudo J. M. Weaver C. M. Crimins J. L. Hunt B.. (2015). Age-related changes to layer 3 pyramidal cells in the rhesus monkey visual cortex. Cereb. Cortex 25, 1454–1468. 10.1093/cercor/bht336 24323499 PMC4428297

[B23] Mancarci B. O. Toker L. Tripathy S. J. Li B. Rocco B. Sibille E.. (2017). Cross-Laboratory analysis of brain cell type transcriptomes with applications to interpretation of bulk tissue data. eNeuro 4:ENEURO.0212-17.2017. 10.1523/ENEURO.0212-17.2017 29204516 PMC5707795

[B24] Moor A. E. Harnik Y. Ben-Moshe S. Massasa E. E. Rozenberg M. Eilam R.. (2018). Spatial reconstruction of single enterocytes uncovers broad zonation along the intestinal villus axis. Cell 175, 1156–1167. 10.1016/j.cell.2018.08.063 30270040

[B25] Newton D. F. Oh H. Shukla R. Misquitta K. Fee C. Banasr M.. (2020). Chronic stress induces co-ordinated cortical microcircuit cell type transcriptomic changes consistent with altered information processing. bioRxiv 2020.08.18.249995. 10.1101/2020.08.18.249995 34861977

[B26] Nizzardo M. Taiana M. Rizzo F. Aguila Benitez J. Nijssen J. Allodi I.. (2020). Synaptotagmin 13 is neuroprotective across motor neuron diseases. Acta Neuropathol. 139, 837–853. 10.1007/s00401-020-02133-x 32065260 PMC7181443

[B27] Okaty B. W. Sugino K. Nelson S. B. (2011). A quantitative comparison of cell-type-specific microarray gene expression profiling methods in the mouse brain. PLoS ONE 6:e16493. 10.1371/journal.pone.0016493 21304595 PMC3029380

[B28] Pereira M. Birtele M. Shrigley S. Benitez J. A. Hedlund E. Parmar M.. (2017). Direct reprogramming of resident NG2 glia into neurons with properties of fast-spiking parvalbumin-containing interneurons. Stem Cell Rep. 9, 742–751. 10.1016/j.stemcr.2017.07.023 28844658 PMC5599255

[B29] Progatzky F. Dallman M. J. Lo Celso C. (2013). From seeing to believing: labelling strategies for *in vivo* cell-tracking experiments. Interface Focus 3:20130001. 10.1098/rsfs.2013.0001 23853708 PMC3638420

[B30] Qiao H. Li M.-X. Xu C. Chen H.-B. An S.-C. Ma X.-M. (2016). Dendritic spines in depression: what we learned from animal models. Neural Plast. 2016:8056370. 10.1155/2016/8056370 26881133 PMC4736982

[B31] Ritchie M. E. Phipson B. Wu D. Hu Y. Law C. W. Shi W.. (2015). limma powers differential expression analyses for RNA-sequencing and microarray studies. Nucleic Acids Res. 43:e47. 10.1093/nar/gkv007 25605792 PMC4402510

[B32] Rocco B. R. Oh H. Shukla R. Mechawar N. Sibille E. (2017). Fluorescence-based cell-specific detection for laser-capture microdissection in human brain. Sci. Rep. 7:14213. 10.1038/s41598-017-14484-9 29079825 PMC5660154

[B33] Rosenblatt J. Stein J. (2020). RRHO: Inference on Agreement Between Ordered Lists [Internet]. Bioconductor Version: Release (3.11). Available online at: https://bioconductor.org/packages/RRHO/ (accessed November 30, 2020).

[B34] Scala F. Kobak D. Bernabucci M. Bernaerts Y. Cadwell C. R. Castro J. R.. (2020). Phenotypic variation of transcriptomic cell types in mouse motor cortex. Nature 1–7. 10.1038/s41586-020-2907-3 33184512 PMC8113357

[B35] Shukla R. Prevot T. D. French L. Isserlin R. Rocco B. R. Banasr M.. (2019). The relative contributions of cell-dependent cortical microcircuit aging to cognition and anxiety. Biol. Psychiatry. 85, 257–267. 10.1016/j.biopsych.2018.09.019 30446205

[B36] Soneson C. Delorenzi M. (2013). A comparison of methods for differential expression analysis of RNA-seq data. BMC Bioinformatics 14:91. 10.1186/1471-2105-14-91 23497356 PMC3608160

[B37] Stockmeier C. A. Mahajan G. J. Konick L. C. Overholser J. C. Jurjus G. J. Meltzer H. Y.. (2004). Cellular changes in the postmortem hippocampus in major depression. Biol. Psychiatry 56, 640–650. 10.1016/j.biopsych.2004.08.022 15522247 PMC2929806

[B38] Stuart T. Butler A. Hoffman P. Hafemeister C. Papalexi E. Mauck W. M.. (2019). Comprehensive integration of single-cell data. Cell 177, 1888–1902. 10.1016/j.cell.2019.05.031 31178118 PMC6687398

[B39] Tasic B. Menon V. Nguyen T. N. Kim T. K. Jarsky T. Yao Z.. (2016). Adult mouse cortical cell taxonomy revealed by single cell transcriptomics. Nat. Neurosci. 19, 335–346. 10.1038/nn.4216 26727548 PMC4985242

[B40] Tasic B. Yao Z. Graybuck L. T. Smith K. A. Nguyen T. N. Bertagnolli D.. (2018). Shared and distinct transcriptomic cell types across neocortical areas. Nature 563, 72–78. 10.1038/s41586-018-0654-5 30382198 PMC6456269

[B41] Toker L. Mancarci B. O. Tripathy S. Pavlidis P. (2018). Transcriptomic evidence for alterations in astrocytes and parvalbumin interneurons in subjects with bipolar disorder and schizophrenia. Biol Psychiatry 84, 787–796. 10.1016/j.biopsych.2018.07.010 30177255 PMC6226343

[B42] Tremblay R. Lee S. Rudy B. (2016). GABAergic interneurons in the neocortex: from cellular properties to circuits. Neuron 91, 260–292. 10.1016/j.neuron.2016.06.033 27477017 PMC4980915

[B43] Tripathy S. J. Toker L. Bomkamp C. Mancarci B. O. Belmadani M. Pavlidis P. (2018). Assessing transcriptome quality in patch-seq datasets. Front. Mol. Neurosci. 11:363. 10.3389/fnmol.2018.00363 30349457 PMC6187980

[B44] Verkhratsky A. Rodrigues J. J. Pivoriunas A. Zorec R. Semyanov A. (2019). Astroglial atrophy in Alzheimer's disease. Pflugers Arch. 471, 1247–1261. 10.1007/s00424-019-02310-2 31520182

[B45] Wang Y. Song W. Wang J. Wang T. Xiong X. Qi Z.. (2019). Single-cell transcriptome analysis reveals differential nutrient absorption functions in human intestine. J. Exp. Med. 217:e20191130. 10.1084/jem.20191130 31753849 PMC7041720

[B46] Yuan G.-C. Cai L. Elowitz M. Enver T. Fan G. Guo G.. (2017). Challenges and emerging directions in single-cell analysis. Genome Biol. 18:84. 10.1186/s13059-017-1218-y 28482897 PMC5421338

