# Supplementary figures and images for: Estimating and Correcting for Off-Target Cellular Contamination in Brain Cell Type Specific RNA-Seq Data
Source: Front Mol Neurosci. 2021 Mar 3;14:637143. doi: 10.3389/fnmol.2021.637143 (PMC7966716; doi:10.3389/fnmol.2021.637143)

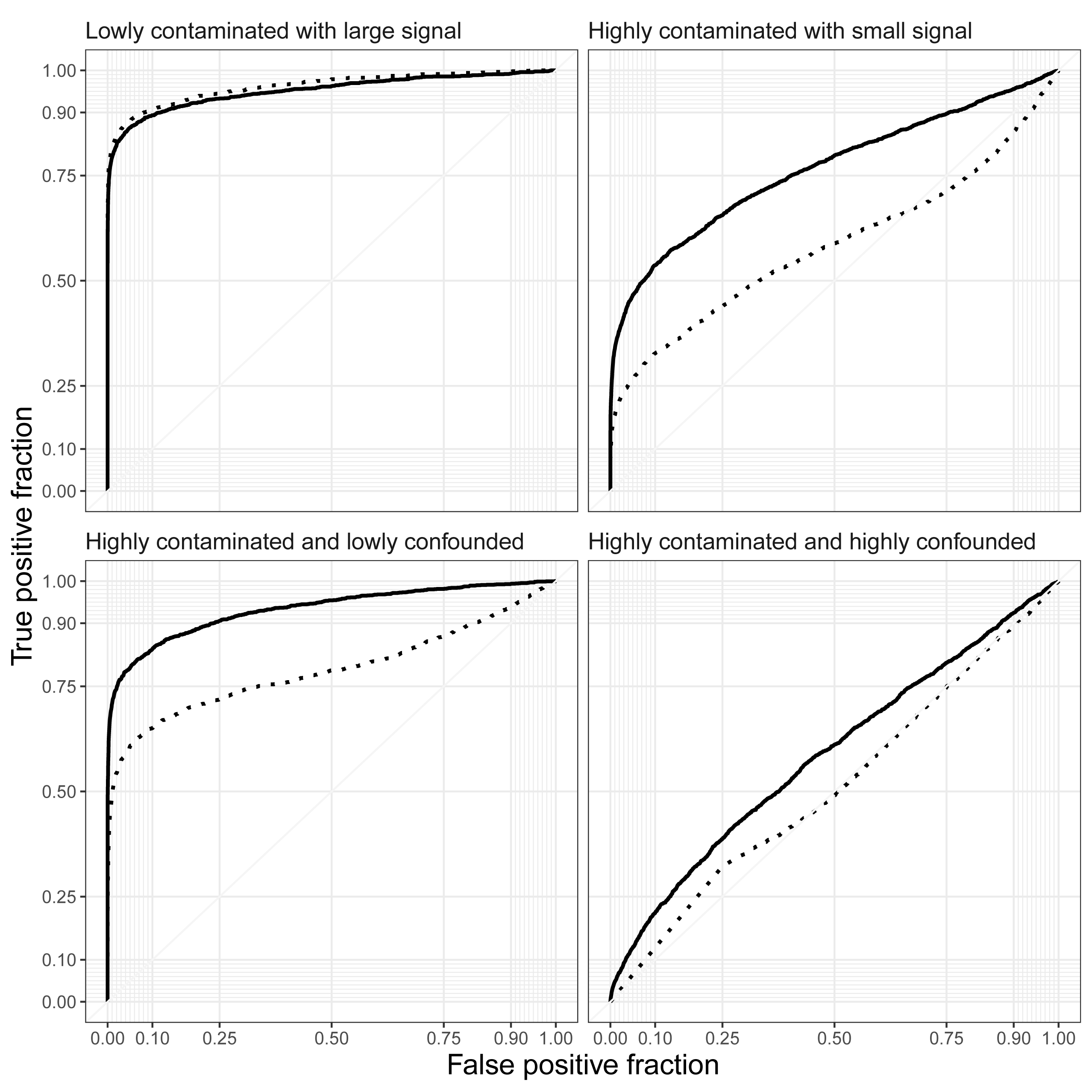

Supplement: Supplementary file 3 [file Image_1.TIF]
