# Supplementary material for: Estimating and Correcting for Off-Target Cellular Contamination in Brain Cell Type Specific RNA-Seq Data
Source: Front Mol Neurosci. 2021 Mar 3;14:637143. doi: 10.3389/fnmol.2021.637143 (PMC7966716; doi:10.3389/fnmol.2021.637143)
Supplement: Supplementary file 4 [file Data_Sheet_1.DOCX]

Supplementary Material

**Figure S1. Differential expression analysis on synthetic data is made more accurate using contamination estimates.** Receiver Operating Characteristic (ROC) curves for the differential expression analysis on four synthetic datasets with varying parameters, both using contamination coefficients in the model (solid lines) and not (dashed lines). After including contamination coefficients as covariates in the differential expression model, improvement is seen in nearly every simulation.

**Table S1. Marker genes calculated on reference data used in this analysis.** Cell type specific marker genes calculated using Seurat on the reference single cell RNA-seq data.

**Table S2. Differentially expressed genes.** Differential expression analysis output for the “Aging” and “Stress” datasets. These were calculated using DESeq2 with and without the top four largest contamination coefficients as covariates in the linear model.
